# Th17: A New Participant in Gut Dysfunction in Mice Infected with *Trichinella spiralis*

**DOI:** 10.1155/2009/517052

**Published:** 2009-11-18

**Authors:** Yu Fu, Wenfeng Wang, Jingjing Tong, Qi Pan, Yanqing Long, Wei Qian, Xiaohua Hou

**Affiliations:** Division of Gastroenterology, Union Hospital, Tongji Medical College, Huazhong University of Science and Technology, Jiefang Avenue 1277, Wuhan 430022, China

## Abstract

*Trichinella spiralis* infection in rodents is a well-known model of intestinal inflammation associated with hypermotility. Our aim was to elucidate if Th17 cells were involved in the development of gastrointestinal hypermotility in this experimental model. Intestinal inflammation was observed by hematoxylin-eosin (HE) staining. Jejunal smooth muscle contractility was investigated in response to acetylcholine (Ach). The effects of IL-17 on jejunum smooth muscle contractility were explored. Flow cytometry was used to analyze the proportion of Th17 cells in jejunum. The levels of IL-17, IL-23, and TGF-*β*1 in jejunum were measured by Western blot. Our results showed that the inflammation in jejunum was severe at 2 weeks postinfection (PI), which was not discernible at 8 weeks PI. Jejunal smooth muscle contractility was increased at 2 weeks PI and kept higher at 12 weeks PI. The proportion of Th17 cells and the expression of IL-17 were upregulated in jejunum at 2 weeks PI and normalized at 8 weeks PI. When jejunual smooth muscle strips were cultured with IL-17, contractions elicited by Ach were enhanced in a concentration-dependent manner. Our data suggest that Th17 cells are increased during acute infection with *Trichinella spiralis* and IL-17 may contribute to jejunal muscle contractility in mice.

## 1. Introduction

Intestinal motor alterations are associated with clinical symptoms such as diarrhea, constipation, and abdominal pain. Studies demonstrated that visceral hypersensitivity and persistently altered intestinal muscle dysfunction existed in mice infected with *Trichinella spiralis* [1, 2]. In fact, the adaptive response to intestinal parasites has been suggested as a paradigmatic defense response of the intestine against external pathogens. For these reasons, experimental parasite infection has been commonly used as model to understand pathogenesis of intestinal dysfunction [3–5].

The immune changes in *Trichinella spiralis* infection appear to be T helper (Th) cell dependent, as have been shown to be prevented by cyclosporine [6]. According to their capability of producing cytokines, Th cells were classified as three distinct subsets, Th1, Th2, and Th17. Characteristically, Th1-type cells produce interleukin (IL)-12 and IFN-*γ*; Th2-type cells synthesize IL-4, IL-5, IL-6, IL-9, and IL-10. Th17-type cells, newly discovered subset of CD4 + effort T cells, produce IL-17 distinctively.

 Although studies have been performed on the roles of Th2 cells and related cytokines in intestinal dysfunction during infection of *Trichinella spiralis* [7, 8], it is unknown about the functionality of Th17 in this process. Previously, TGF-*β*_1_ has been commonly considered as an anti-inflammatory cytokine but now found to be critical in the differentiation of Th17 cells [9]. Recent work by D. Yen showed that IL-23 stimulated Th17 to generate the proinflammatory mediator IL-17, which is important to maintain the chronic intestinal inflammation [10]. The aim of this study is to analyze the roles of Th17 cells and related cytokines in gut dysfunction in mice infected with *Trichinella spiralis*. 

## 2. Materials and Methods

### 2.1. Mice

Studies were performed on male inbred National Institute of Health (NIH) mice obtained from the Institution of Biological Product (Wuhan, China) and used between 6 and 8 weeks of age. Mice were bred in an accredited facility at the Institute for Animal Health maintained at 23-24°C in temperature and a light-dark cycle of 12-12 h (lights on at 7 Am). Experiments were approved by the Ethics Committee of Tongji Medical College.

### 2.2. Trichinella Infection

*Trichinella spiralis* cultures were originated in the Department of Parasitology at the University of Huazhong Science and Technique. The colony was maintained through infection in Sprague-Dawley rats. Larvae of *Trichinella spiralis* were obtained from the rats infected for more than 30 days. The infected rats were killed and skinned. The muscles containing the encysted larvae were finely minced and digested in 0.5% pepsin A (Sigma-Aldrich) and 0.5% of HCL at 37°C for 5 houres. The isolated infective larvae were washed several times with 0.85% NaCl and suspended in 2% of Agar (Sigma-Aldrich). Mice were infected by the oral administration of 300 larvae in 0.2 mL of Agar following a modified method described by Wheatcroft J [11] 

### 2.3. Time Course of Response to Infection

 NIH mice were sacrificed at 2 weeks, 8 weeks, and 12 weeks PI. Each group had 6–8 mice. Following studies were conducted at each time point: histological changes of jejunum were observed; the jejunal smooth muscle contractility was investigated in response to acetylcholine; the levels of IL-17, IL-23, and TGF-*β*_1_ and the proportions of Th17 cells subset in jejunum were also analyzed. 

### 2.4. Histological Study

 Samples of jejunum were obtained, fixed for 48 houres in 10% neutral buffered formalin, embedded in paraffin, cut into 5 um sections, and stained with hematoxylin-eosin (H&E) according to standard procedures. 

### 2.5. Tissue Preparation and Organ Culture Procedure

The method used was described in detail by Ohama et al. [12]. Briefly, mice were executed by cervical vertebra disjointing. A 10 cm segment of the jejunum was detached from mesenterium and placed in sterile Hanks' balanced salt solution. Strips were teased along the natural line of cleavage from the longitudinal smooth muscle and then transferred to culture dishes with Medium 199 (Invitrogen) supplemented with penicillin (100 units/mL)/streptomycin (100 ug/mL, Gibco) and L-glutamine (200M, Invitrogen). The culture dishes were incubated at 37°C in an atmosphere of 95% air and 5% CO_2_. The incubation medium was replaced every day. Jejunum longitudinal muscle was preincubated with or without IL-17 (0.1-10 ng/mL, Peprotech) for 2 days. Freshly isolated smooth muscle strips were prepared as described above but not under sterile conditions.

### 2.6. Measurement of Muscle Contractility

 Longitudinal muscle strips were cut by 3 mm × 10 mm and then placed in 10 mL organ bath containing warm (37°C) oxygenated (95% O_2_, 5% CO_2_) Krebs solution. The upper end of each strip was attached to an isometric force transducer (Fort-10, WPI, USA), which was connected to an amplifier. The digitized data were collected by a computer equipped with Acqknowledge 3.7.1 software (BIOPAC system, USA). After an equilibration period of 60 minutes with flushing in every 15 minutes at a load of 0.25 g, the length–tension relationship was recorded. Muscle strips were stretched by load increments of 0.25 g exposed to 10^−6^ M Ach. The degree of applied tension producing the maximum response to Ach was identified as optional tension. The area under curve (AUC; g*·*s) was measured in time intervals of 5 min after Ach addition. The response in different groups was quantified by calculating the AUC when muscles were stretched by application of optional tension.

### 2.7. Isolation of Intestinal Lamina Propria Mononuclear Cells

 Jejunum was thoroughly washed with PBS and then cut into 0.5 cm pieces. The epithelium was removed by incubation with 1 mM DTT (Sigma-Aldrich) and 1 mM EDTA (Sigma-Aldrich) in RPMI 1640 medium supplemented with 5% FCS at 37°C for 30 min with gently shaking. After repeating this step twice, the tissue was cut into smaller pieces and then digested with 1 mg/mL collagenase D (Roch) at 37°C for 90 min. Lamina propria cells were harvested by discontinuous 40/70 percoll gradient (Amersham Biosciences).

### 2.8. Surface and Intracellular Cytokine Staining

 Cells obtained from dissection of lamina propria were incubated for 4 h with 50 ng/mL PMA (Alexis), 750 ng/mL Ionomycin (Sigma-Aldrich), and 10 mg/mL Brefeldin A (Biolegend) in a tissue culture incubator at 37°C. After surfaces staining with the phycoerythrin-conjugated antimouse CD4 antibody (Biolegend), the cells were fixed and permeabilized with Fixation/Permeabilization solution (Biolegend). Then the cells were stained intracellularly with allophycocyanin-conjugated antimouse IL-17 antibody (Biolegend). Cytokine staining was performed as the manufacturer's protocol. Samples were acquired on a LSR II (BD Biosciences) and data were analyzed by FACSDiVa software (BD Biosciences).

### 2.9. Western Blot Analysis

 A total of 80 *μ*g of protein lysates derived from jejunal tissue samples were loaded on 15% SDS-PAGE gels. Membranes were probed overnight at 4°C with antibodies against IL-17A (R&D System), TGF-*β*_1_ (Biovision), IL-23p19 (Santa Cruz), or *β*-Actin (Pierce) antibodies, followed by the appropriate species-specific horseradish peroxidase conjugate (Pierce) and developing in the SuperSignal West Pico Substrate (Pierce). Band intensities were quantitated by the Quantity One 4.6.2 software (BioRad).

### 2.10. Statistical Analysis

 Data are expressed as means ± SD. Statistical significance was calculated with the Kruskal-Wallis or Mann-Whitney test as appropriate using SPSS 11.0 software. The correlation between gut contraction and expression of IL-17 was analyzed using Spearman's rho-test. All *P* value of .05 or less was considered significant.

## 3. Results

### 3.1. Morphology

**Figure 1:**
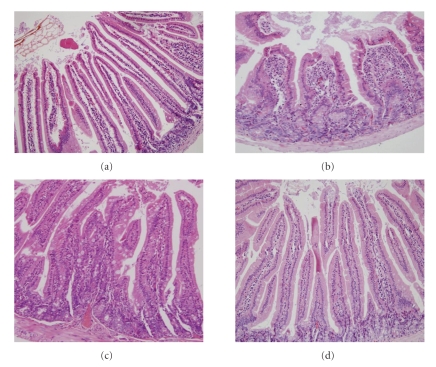
H&E staining of jejunum in (a) control mice and mice infected with T. spiralis at (b) 2 weeks PI, (c) 8 weeks PI, and (d) 12 weeks PI (magnification 200x): the mucosal damage was sever at 2 weeks PI, characterized by hyperemia, swelling, and significant decrease in villus height. Mixed infiltration of neutrophil cells, eosinophil cells, and lymphocytes affected the mucosal and submucosal layers.

 At 2 weeks PI, H&E staining of the jejunum showed hyperemia, swelling, and decrease in villus height. An intense inflammatory response with mixed infiltration of neutrophil cells, eosinophil cells, and lymphocytes affecting the mucosal and submucosal layers was induced by T. Spiralis infection. There was no discernible inflammation presented in the gut at 8 and 12 weeks PI (Figure 1).

### 3.2. Jejunal Smooth Muscle Contraction Response to Acetylcholine

**Figure 2:**
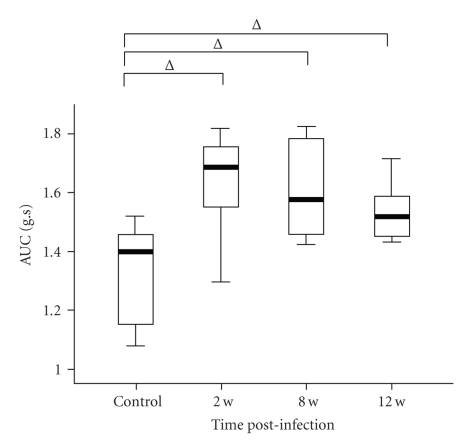
Responses of the jejunal longitudinal muscle to 10^−6^ M Ach-induced contraction. Responses were obtained from tissues stretched when optional tention was applied. The upper and lower whiskers indicate the maximum and minimum values, respectively. Box lines represent the 25th (bottom), 50th (middle), and 75th (top) percentile value; ∆*P* < .05 indicate differences between control and *Trichinella spiralis*-infected mice.

 Increased contractile responses to Ach were noted in longitudinal muscle strips from 2 weeks to 12 weeks PI in the T. Spiralis infected mice (Figure 2). At 2 weeks after infection, the AUC in the infected mice was significantly increased over control when maximum response was generated by longitudinal muscle response to ACh (1.63 ± 0.19 g*·*s versus 1.34 ± 0.18 g*·*s, *P* = .026). Longitudinal muscle contraction response kept higher at 8 weeks PI (1.60 ± 0.17 g*·*s versus control, *P* = .041) and 12 weeks PI (*f* ± 0.10 g*·*s versus control, *P* = .026). 

### 3.3. The Proportion of Th17 Cells in Mucosal Lamina Propria

**Figure 3:**
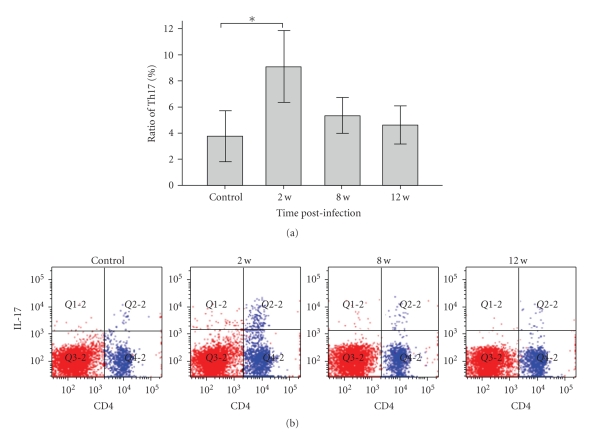
(a) Percentages of Th17 cells in jejunum of control and infected mice at different time points. The proportion of Th17 at 2 weeks was significant higher than control, but normalized at 8 and 12 weeks. *P < .01 indicate differences between control and *Trichinella spiralis*-infected mice. (b) Representative flow cytometric analyses in jejunum of control and infected mice at different time points.

 In jejunum, a significant proportional increase was observed for IL-17 producing cells in the infected mice at 2 weeks PI compared with controls (9.13 ± 2.73 versus 3.78 ± 1.97, *P* < .01) and normalized at 8 weeks (5.38 ± 1.37) and 12 weeks (4.68 ± 1.48) (Figure 3).

### 3.4. Cytokine Expression

**Figure 4:**
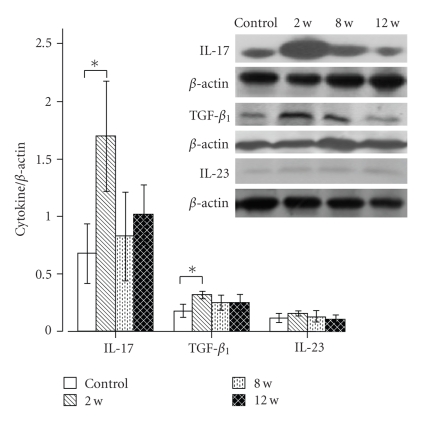
Expressions of IL-17, IL-23, and TGF-*β*_1 _in jejunum in control and infected mice at various time points. Protein expression was assessed by western blotting. *P < .01 indicate differences between control and *Trichinella spiralis*-infected mice.

 IL-17 is the key cytokine secreted by Th17 cells characteristically [13]. To explore whether Th17 cells take effect during infection, we analyzed the content of IL-17 in jejunum at various time points. The results showed that the expression of IL-17 was significantly elevated (*P *< .01) at 2 weeks PI and turned to normal thereafter (Figure 4). 

 Though studies have showed that TGF-*β*_1_ and IL-23 take part in the differentiation and expansion of Th17 cells [9, 10], but it is not clear whether TGF-*β*_1_ and IL-23 are involved in the inflammation in mice infected with *Trichinella spiralis*, so the levels of these two cytokines in the gut were studied at each time point. In jejunum, a higher level of TGF-*β*_1_ in infected mice was noted at 2 weeks PI compared with uninfected mice (0.31 ± 0.03 versus 0.17 ± 0.05,* P *<.01), and normalized at 8 and 12 weeks PI (0.26 ± 0.07 and 0.24 ± 0.07, resp.) (Figure 4). The expressions of IL-23 in the jejunum at 2, 8, and 12 weeks PI were 0.16 ± 0.02, 0.14 ± 0.04, and 0.11 ± 0.03, respectively, and no significant changes were found compared with control during infection (Figure 4).

### 3.5. Association between Gut Contraction and Expression of IL-17

**Figure 5:**
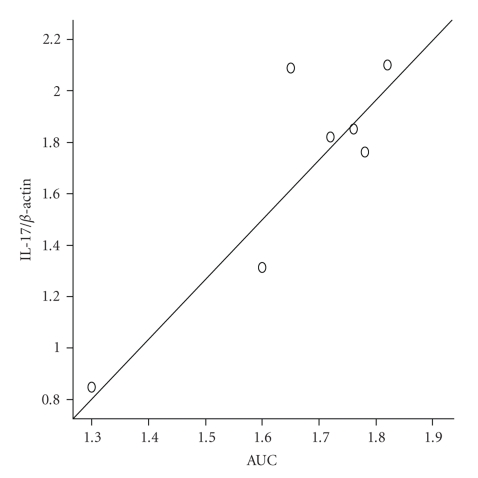
Correlation of IL-17 expression and muscle contraction assessed by responses of the longitudinal muscle to 10^−6^ M Ach-induced contraction in jejunum. IL-17 expression is associated significantly with the contraction of jejunal longitudinal muscle (*r * = 0.762, *P* = .028) at 2 weeks PI.

 To explore whether the jejunum hypercontractility in the infected mice was affected by the expression of IL-17, the relationship of jejunal muscle contraction and expression of IL-17 was analyzed. We found that the expression of IL-17 in jejunum was correlated significantly with jejunum longitudinal muscle contraction (*r* = 0.773, *P *= .039) at 2 weeks PI (Figure 5), while the correlations could not be observed at 8 or 12 weeks PI.

### 3.6. IL-17-Induced Muscle Hypercontractility

**Figure 6:**
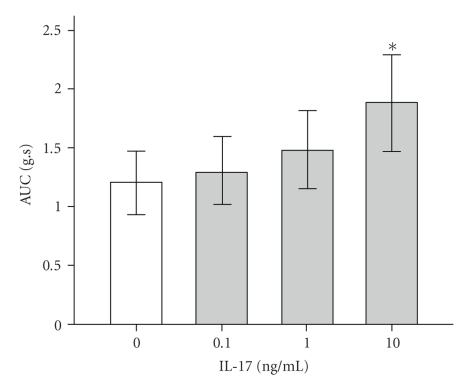
Concentration-dependent change in contractions induced by acetylcholine (10^−6^ M) shows the effects on acetylcholine-induced contractions of various concentrations of IL-17 (0.1–10 ng/mL) in a 2-day culture. *P < .01 indicate significantly different from control (white column).

To determine the effect of IL-17 on muscle contractility, jejunum longitudinal muscle isolated from normal mice was preincubated with IL-17 (0.1–10 ng/mL) for 2 days and then stimulated by 10^−6^ M Ach. In the presence of IL-17, contractions elicited by Ach were enhanced in a concentration-dependent manner. The concentration dependence of IL-17 indicated that 0.1 and 1 ng/mL IL-17induced less increase of contractile forces in jejunum longitudinal smooth muscles cultured for 2 days, while 10 ng/mL IL-17 promoted the contractile significantly (Figure 6).

## 4. Discussion

A number of parasite infections in rodents cause intestinal inflammation, such as *Trichinella spiralis*. During a relatively brief intestine stage for 1-2 weeks, adult female worms release newborn larvae that rapidly enter mesenteric venule [14], disseminate throughout the host, and eventually enter skeletal muscle to encyst at about 1 month PI, which is known as muscle stage. In our study, histology investigation showed that the presence of adult worms and larvae in the jejunal mucosa cause a severe inflammatory response at 2 weeks PI, which persists until eviction of the parasite. After the larvae transferred to skeletal muscle, no discernible inflammation was presented at 8 and 12 weeks PI. 

 Parasites have provided excellent models for studying the intestine dysfunction during and after infection of pathogens. Intestinal muscle hypercontractility can be observed at the early stage of infection and last for a long time after intestinal inflammation recovered [1, 2]. Previous studies performed on NIH Swiss mice showed that contractile response of jejunum longitudinal muscle strips was remarkably increased during acute *Trichinella spiralis* infection [1]. By 21 days postinfection, the adult worms leave the host and the acute inflammation normalizes, but functional alterations of the small intestine persist for at least a further 21 days [15]. In our study, the NIH mice were used, which are genetically closely related to but not identical to the NIH Swiss mice and behave immunologically very similarly to the NIH Swiss mice [16]. It should be recognized that we not only have proven that NIH mice infected with *Trichinella spiralis* showed the similar motility abnormalities which others have demonstrated using this model in NIH Swiss mice [1] but also have proven that the hypercontractility lasted for 12 weeks PI. 

 Recent studies demonstrated that altered intestinal motility in infected mice was associated with the increased T cell in gut, which can be reversed by a corticosteroid treatment [15]. T cells also mediate the hypercontractile state of muscle during *Trichinella spiralis* infection [17, 18]. Furthermore, reports show that Th2 cytokines can induce muscle hypercontractility by a direct action on smooth muscle cell [8], while data about Th17 cells are not available. We analyzed the proportion of Th17 cells isolated from mucosal lamina propria and the expression of IL-17 in jejunum in mice infected with *Trichinella spiralis*. The results showed that the proportion of Th17 cells and the level of IL-17 were increased at 2 weeks PI compared with the controls and recovered at 8 weeks PI. Meanwhile, we found that the expression of IL-17 correlated with the jejunal smooth muscle contraction at 2 weeks PI, which implied that the hypercontractility of intestine smooth muscle was affected by the content of IL-17 in intestine. To confirm the effect of IL-17 on muscle contraction, we investigated the jejunum longitudinal smooth muscle contractility preincubated with or without IL-17. We found that IL-17induced increase of contractile forces in a concentration-dependent manner. Similar finding was observed in another study that IL-17 also has a role in airway hypersensitivity responses, such as asthma and chronic obstructive pulmonary disease [19, 20], which are associated with the increased number of neutrophils and linked to IL-17. But how IL-17 alerts smooth muscle contraction, by regulating excitation-contraction coupling, by inducing other cytokines, or by other means? The mechanism underlying the influence of IL-17 on the contractility of intestine smooth muscle in mice during infection needs to be further investigated. It should be noted that the jejunal hyper contractility lasted for 12 week PI when IL-17 had normalized, which suggested that other cytokines or cells might work in this period of time. 

Weinstock JV reported that helminth infection down-regulates IL-17 production by lamina propria mononuclear cells (LPMCs) and mesenteric lymph node cells at 2 weeks PI [21]. In our study, increased number of Th17 and content of IL-17 in jejunum were observed at 2 weeks PI and normalized thereafter. It has been shown that the time course during infection varies in different pathogen strains of mice and using different doses of larvae [22, 23]. This study used C57BL/6 mice infected with 150 H. polygyrus [21], while our study used NIH mice infected with 300 T. spiralis. Therefore the results may be different. 

 Polarization of T cells subset can be influenced by several factors, including the cytokine microenvironment, differential antigen processing, and antigen characteristics. TGF-*β*_1_ is a pleiotropic cytokine made by multiple cells types [24], which has been in the spotlight because of its emerging roles in the differentiation of Th17 cells from naive T cells [9]. TGF-*β*_1_ not only has a critical function as an antagonist of Th1 development affecting IFN-*γ* as well as T-bet [25], but also interferes with Th2 differentiation [26], thus allowing the diversion to IL-17 T cell differentiation. In our study, the level of TGF-*β*1 in jejunum was increased at 2 weeks PI, in accompany with the upregulating of IL-17 and Th17 cells and then recovered at 8 weeks PI. The results showed that Th17 cells might be induced by TGF-*β*_1_ but not sustained during *Trichinella spiralis* infection. 

 IL-23 is expressed in the intestine in various models of intestinal inflammation [27, 28], which could act by reinforcing the Th17 response to form IL-23-Th17 axis in colitis, although it is not required during the differentiation of Th17 [29]. We also observed the change of IL-23 expression in the jejunum of the *Trichinella spiralis* infected mice at different time points. It is interesting that there was no significant change in the level of IL-23 in the jejunum of infected mice, which indicated that the function of IL-23 in nematode infection is not as important as it does in intestine inflammation induced by IL-10 knockout [10] or pathogenic CD4 T-cell transfer [30]. 

In summary, the present study demonstrated that Th17 cells influenced the intestine smooth muscle contractility during intestinal infection with *Trichinella spiralis*. TGF-*β*1 might induce differentiating of Th17 cells during infection, while IL-23 was not involved in this process. These results not only have implications for host defense against nematodes but also may have broader implications for clinical gastroenterology.
